# Residual diabetic foot osteomyelitis after surgery leads to poor clinical outcomes: A systematic review and meta‐analysis

**DOI:** 10.1111/wrr.13215

**Published:** 2024-10-07

**Authors:** Mario C. Reyes, Mehmet A. Suludere, Arthur N. Tarricone, Tehreem Sajjad, Tyler L. Coye, Matthew J. Sideman, Lawrence A. Lavery

**Affiliations:** ^1^ Department of Plastic Surgery University of Texas Southwestern Medical Center Dallas Texas USA; ^2^ Department of Orthopedic Surgery University of Texas Health Science Center San Antonio Texas USA; ^3^ Department of Vascular Surgery Baylor University College of Medicine Houston Texas USA; ^4^ Department of Surgery University of Texas Health Science Center San Antonio Texas USA

**Keywords:** amputation, diabetes, foot ulcer, infection, neuropathy, osteomyelitis

## Abstract

The aim of this meta‐analysis is to compare the clinical outcomes in patients with and without residual osteomyelitis (ROM) after surgical bone resection for diabetic foot osteomyelitis (DFO). We completed a systematic literature search using PubMed, Scopus, and Embase using keywords DFO, Residual OM (ROM), and positive bone margins. The study outcomes included wound healing, antibiotic duration, amputation, and re‐infection. Five hundred and thirty patients were included in the analysis; 319 had no residual osteomyelitis (NROM), and 211 had ROM. There was not a significant difference in the proportion of wounds that healed 0.6 (*p* = 0.1, 95% confidence intervals [95% CI] 0.3–1.3). The risk of infection was 2.0 times higher (OR = 2.0, *p* = 0.02, 95% CI 1.1–3.4), and the risk of amputation was 4.3 times higher (OR = 4.3, *p* = 0.0001, 95% CI 2.4–7.6) in patients with ROM. Patients with ROM received antibiotics significantly longer. The mean difference was 16.3 days (*p* = 0.02, 95% CI 11.1–21.1).

**Abbreviations**
95% CI: 95% confidence intervals
DFO: diabetic foot osteomyelitis
IDSA: Infectious Disease Society of America
IWGDF: International Working Group on the Diabetic Foot
NOS: Newcastle‐Ottawa Scale
NROM: no residual osteomyelitis
PAD: peripheral arterial disease
PRISMA: Preferred Reporting Items for Systematic Reviews and Meta‐Analysis
ROM: residual osteomyelitis

## 1 INTRODUCTION

Diabetic foot osteomyelitis (DFO) is a challenging pathology, comprising 60% of patients with diabetic foot infections admitted to hospital.^1^, ^2^, ^3^ The treatment of DFO consists of medical management, surgical resection/amputation, or a combination of the two treatment approaches. Patients with DFO are more likely to require surgery and have higher rates of re‐infection, hospitalisation, amputation, and prolonged antibiotic exposure compared with people with soft tissue infections.^3^, ^4^ The 2012 Infectious Disease Society of America (IDSA)^5^ and the 2023 the International Working Group on the Diabetic Foot (IWGDF) and IDSA guidelines^6^ recommend that when surgery was required for osteomyelitis, proximal bone margins should be obtained, and if the margins do not show residual osteomyelitis (ROM), the duration of post‐surgery antibiotic treatment could be reduced to 2–5 days. There have been several retrospective studies that have compared clinical outcomes among diabetic patients with and without ROM after surgery. The aim of this meta‐analysis is to compare the clinical outcomes in patients with and without ROM after surgical treatment for DFO.

## 2 METHODS

### 2.1 Data sources and searches

This systematic review and meta‐analysis was carried out in accordance with the Preferred Reporting Items for Systematic Reviews and Meta‐Analysis (PRISMA) statement.^7^ A literature search of PubMed, Scopus, and Embase, using Medical Subject (MeSH), and Boolean operations were employed in the search strategy and the final search was conducted on 17 August 2023. A combination and variation of the terms (diabetic foot osteomyelitis) AND (diabetic foot osteomyelitis AND residual osteomyelitis OR positive bone margins). For each database, a specific search was generated and converted accordingly. We included only studies published in the English language. No institutional review board approval was required for the current study. This systematic review and meta‐analysis is registered with the Research Registry (reviewregistry1689).

### 2.2 Inclusion and exclusion criteria

We included only studies that compared surgical patients with and without ROM in patients with diabetic foot infection. ROM was defined as a pathology‐positive bone biopsy or a positive culture after surgical resection of infected bone. A clean bone margin (surgical sites with no residual bone infection) was defined as a pathology‐negative or culture negative bone biopsy. Negative pathology was defined as no report of osteomyelitis in the pathology report. Negative cultures were defined as a culture from the proximal resected margin with no bacterial growth at the time of final surgery. Antibiotic duration included oral and parenteral antibiotics as reported in respective studies. Local antibiotic use was not included in the duration. Studies with <10 participants were excluded. We did not impose a restriction on the year of publication. We imposed no restrictions on publication status. Excluded studies included case reports, basic science research, non‐human populations, non‐English translatable studies, systematic reviews, and meta‐analyses. Two independent reviewers (T.L.C. and M.C.R.) selected relevant studies with any disagreements resolved by a third reviewer A.T. A further reference list was also created to capture articles that were not found during the initial database searches but were relevant to this study. Where a consensus was not reached, disagreements were resolved through discussion.

### 2.3 Data extraction and critical appraisal

M.C.R. independently extracted data, which T.L.C. compared with the original citation. Disagreements were resolved through discussion. Extracted data from eligible studies included: first author, publication year, country, peripheral arterial disease (PAD), number of wounds healed, amputation, study size, mean age, percentage of males, re‐infections, and antibiotic duration (Tables 1 and 2). Re‐ulceration (*n* = 1) and time to heal (*n* = 2) data were collected but there were not enough studies for analysis. The papers were assessed for quality using the Newcastle‐Ottawa Quality Assessment Scales. M.C.R. and T.S. independently scored each study and discussed reported differences.

**TABLE 1:** Summary of studies that report surgical bone resection with and without residual osteomyelitis.

Study	Year	Study design	Country	Average F/U (months)	*N*	Age (years)	Male %	No. of PAD
Aragon‐Sanchez	2023	Prospective	Costa Rica	20.8	93	59.5 ± 12.6	77.4	19
Weng	2023	Retrospective	United States	12.0	92	53.38 ± 10.5	58.6	34
Aragon‐Sanchez	2021	Retrospective	Costa Rica	7.2	28	58.1 ± 11.8	78.6	9
Johnson	2019	Retrospective	United States	12.0	66	NR	93.9	27
Schmidt	2019	Prospective	United States	12.0	72	56.9 ± 12.8	81.9	NR
Beieler	2012	Retrospective	United States	26.2	50	NR	88	10
Atway	2012	Retrospective	United States	9.1	27	56.74 ± 11.42	66.7	10
Kowalski	2011	Retrospective	United States	NR	111	63.0 ± 14	75	NR

Abbreviation: PAD, peripheral arterial disease.

**TABLE 2:** Outcomes from published papers in patients with and without ROM.

Study	Year	Number of subjects		Wounds healed		Amputation		Re‐infection		Antibiotic duration	
		ROM	No ROM	ROM	No ROM	ROM	No ROM	ROM	No ROM	ROM	No ROM
Aragon‐Sanchez	2023	61	31	59	30	10	0	13	6	NR	NR
Weng	2023	35	57	NR	NR	12	8	NR	NR	30 ± 15.0	18 ± 15.0
Aragon‐Sanchez	2021	7	21	7	21	0	0	1	2	NR	NR
Johnson	2019	18	48	10	29	NR	NR	8	19	37.6 ± 24.1	17.7 ± 29.6
Schmidt	2019	9	63	NR	NR	0	0	NR	NR	32 ± 36.0	23.69 ± 18.5
Beieler	2012	31	11	NR	NR	12	3	12	3	43	19
Atway	2012	11	16	8	13	3	0	NR	NR	24.81 ± 15.48	10.44 ± 11.64
Kowalski	2011	39	72	NR	NR	17	11	11	6	19	14

Abbreviations: NROM, no residual osteomyelitis; ROM, residual osteomyelitis.

### 2.4 Data analysis

**FIGURE 1:**
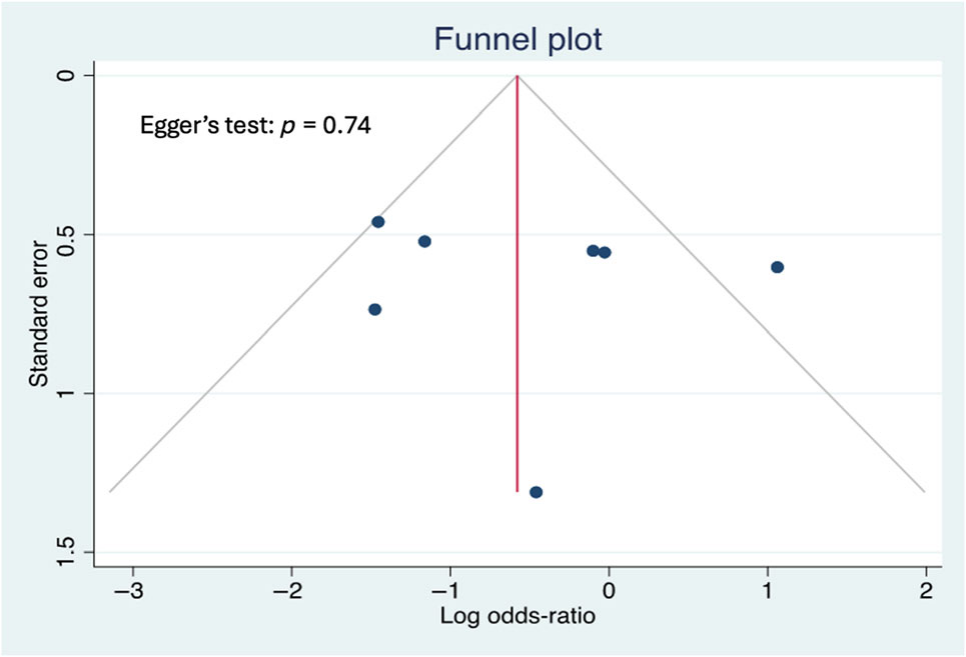
Funnel Plot measuring publication bias Egger's test indicates that there is no strong evidence of publication bias in the meta‐analysis. The high *p*‐value (0.74) suggests that the observed results are likely not significantly skewed by a tendency to publish studies with positive or statistically significant findings.

The primary outcome (wound healing rate) and secondary outcomes (amputation, re‐ulceration, re‐infection, and antibiotic duration) were reported with 95% confidence intervals (95% CI). For variables with low heterogeneity a fixed‐effects model was used. For variables with moderate or high heterogeneity a random‐effects model was chosen to account for the variability among studies. To quantify heterogeneity, we utilised Higgin's & Tompson's *I* ^2^ statistic.^8^ Low heterogeneity was defined as *I* ^2^ < 50%, moderate heterogeneity as *I* ^2^ = 50%–75%, and high heterogeneity as *I* ^2^ > 75%. Data analysis was done using R version 3.6.3 (R Foundation for Statistical Computing, Vienna, Austria), using the meta and metafor package. Funnel plots and Egger's test were used to evaluate publication bias. Egger's test revealed no publication bias (Figure 1).

## 3 RESULTS

### 3.1 Study characteristics and quality

**FIGURE 2:**
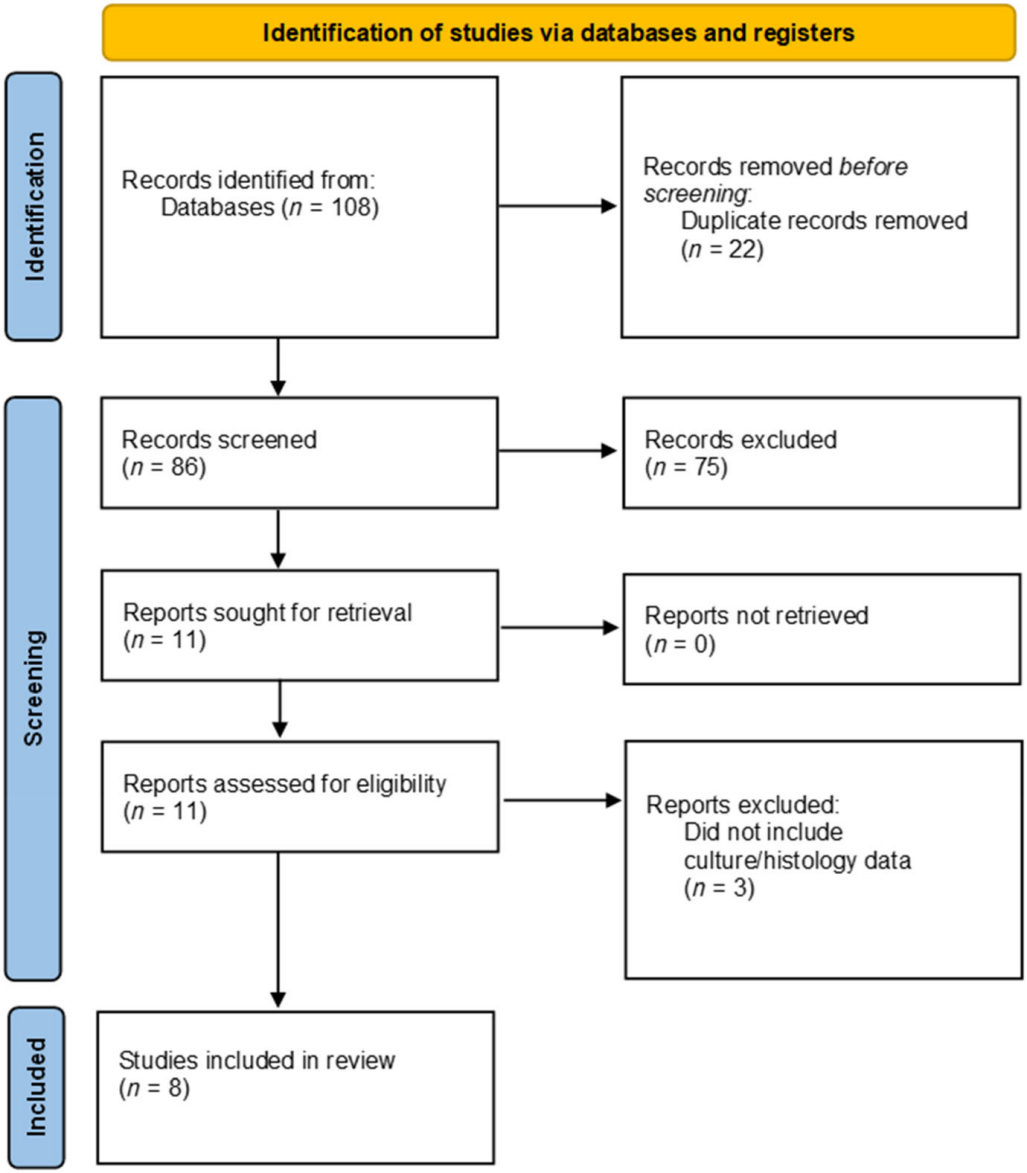
Preferred Reporting Items for Systematic Reviews and Meta‐Analysis (PRSIMA) flow chart of search process. The figure presents the records extracted from three different scientific databases and registrars: PubMed, Scopus, and Embase. All articles were screened based on the inclusion/exclusion criteria and 11 studies were evaluated in full review. Three studies were excluded from this list, yielding eight papers for meta‐analysis.

Eighty‐six citations were identified after the removal of duplicates. Following the title and abstract screening, 11 citations were assessed for full‐text eligibility. Of these, three studies were excluded which did not include histopathology or microbiological culture of bone margins in patients with diabetic foot infection. This resulted in eight studies included in the final analysis. See Figure 2 for PRISMA diagram. Five hundred and thirty patients were included in the analysis. Patient characteristics are summarised in Table 1. Of these, 319 had no residual osteomyelitis (NROM) and 211 had ROM at the surgical margin. There was a mean follow‐up time of 14.1 months (95% CI 9.4–18.8). The mean age was 57.9 years (95% CI 55.3–60.5). The mean percentage of males was 77.5% (95% CI 69.7–85.3). The reported NOS scores range was 4–7 out of 9 as given in Table 3.

**TABLE 3:** Newcastle‐Ottawa scoring table.

Study	Selection/4	Comparability/2	Outcome/3	Total/9
Aragon‐Sanchez et al.^13^	3		3	6/9
Weng et al.^11^	4		2	6/9
Aragon‐Sanchez et al.^14^	3		3	6/9
Johnson et al.^9^	3		3	6/9
Schmidt et al.^10^	3		3	6/9
Kowalski et al.^15^	4		1	5/9
Beieler et al.^16^	4		3	7/9
Atway et al.^12^	3		1	4/9

### 3.2 Antibiotic duration

**FIGURE 3:**
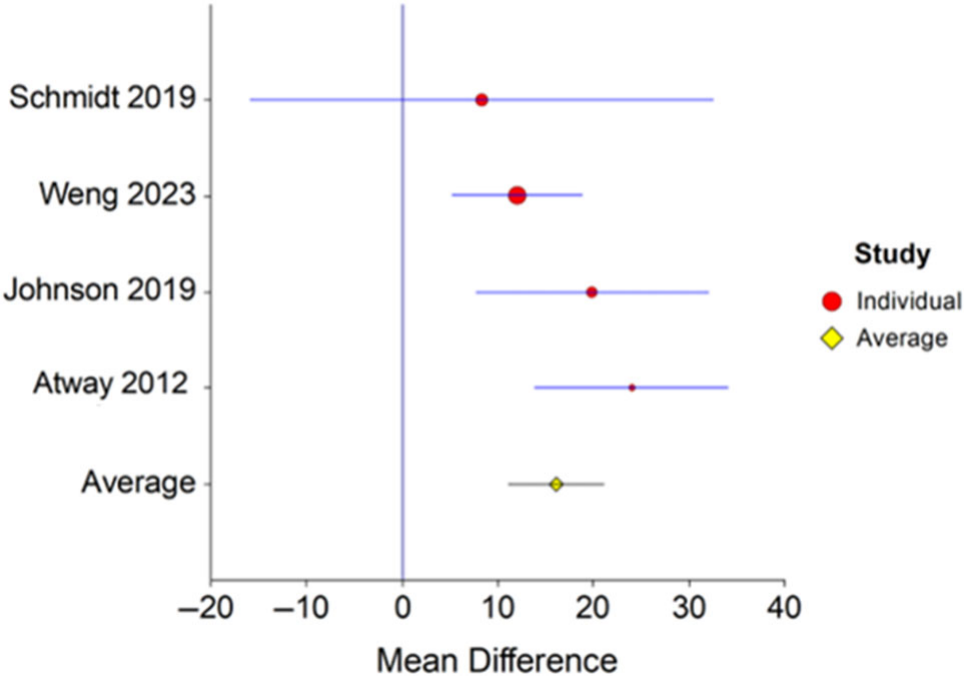
Forest plot of antibiotic duration in patients with and without residual osteomyelitis. Forest plot depicting the mean difference in antibiotic duration in patients with and without residual osteomyelitis following surgical resection. Patients with residual osteomyelitis had longer antibiotic treatment with an average mean difference across all studies of 16.3 days (*p* = 0.02, 95% CI 11.1–21.1). There was no evidence of heterogeneity, Cochrane *Q* = 4.5 (*p* = 0.2) and *I* ^2^ = 33.2 (95% CI 0–76.3).

Only four studies^9^, ^10^, ^11^, ^12^ reported means and standard deviations for antibiotic duration which allowed for meta‐analysis. Four studies included 257 pooled participants. Seventy‐three had ROM and 184 had NROM (Figure 3). Patients with ROM were treated with antibiotics significantly longer than people with no residual infection. The mean difference was 16.3 days (*p* = 0.02, 95% CI 11.1–21.1). There was no evidence of heterogeneity, Cochrane *Q* = 4.5 (*p* = 0.2) and *I* ^2^ = 33.2 (95% CI 0–76.3).

### 3.3 Amputation

**FIGURE 4:**
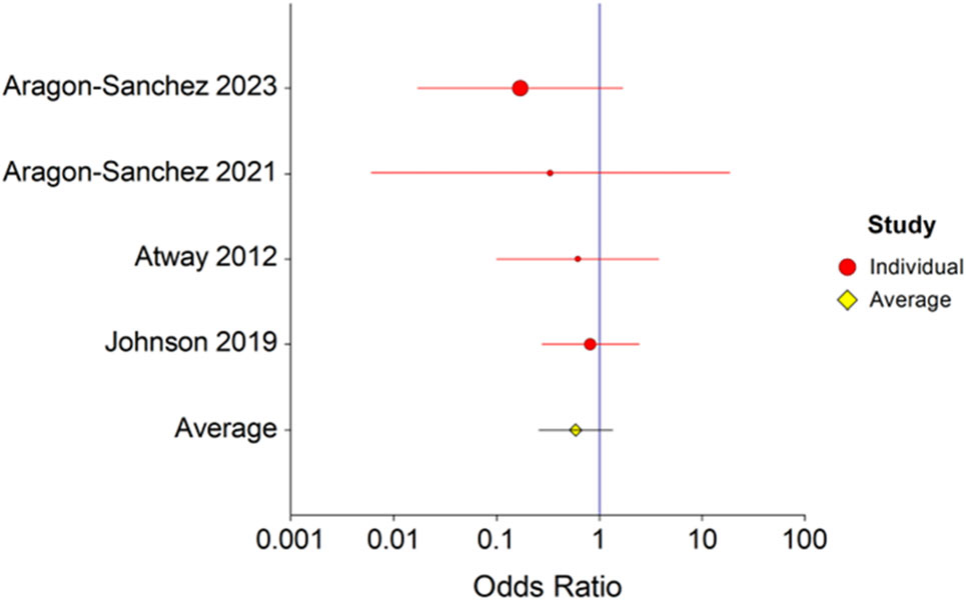
Forest plot of re‐amputation in patients with and without residual osteomyelitis. Forest plot depicting re‐amputation in patients with and without residual osteomyelitis following surgical resection. The risk of amputation was 4.3 times higher when there was residual osteomyelitis, (OR = 4.3, *p* = 0.0001, 95% CI 2.4–7.6). There was no evidence of heterogeneity, Cochrane *Q* = 1.4 (*p* = 0.96) and *I* ^2^ = 0.0 (95% CI 0.0–0.0).

Six studies^10^, ^11^, ^12^, ^13^, ^14^, ^15^, ^16^ with 193 patients with residual bone infection and 271 patients with NROM reported amputation rates (Figure 4). Using a fixed‐effects model, the risk of amputation was 4.3 times higher when there was ROM (OR = 4.3, *p* = 0.0001, 95% CI 2.4–7.6). There was no evidence of heterogeneity, Cochrane *Q* = 1.4 (*p* = 0.96) and *I* ^2^ = 0.0 (95% CI 0.0–0.0).

### 3.4 Wound healing

**FIGURE 5:**
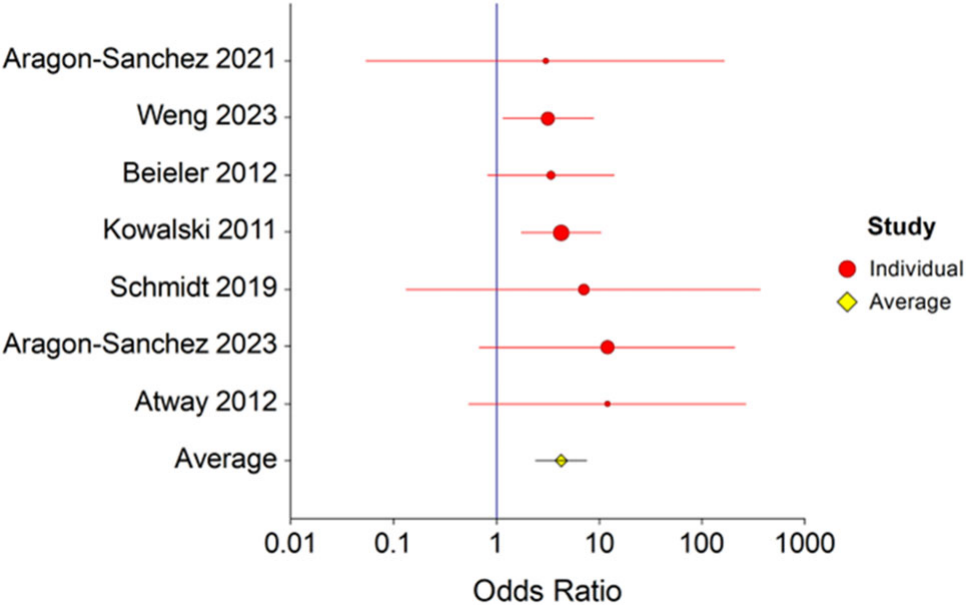
Forest plot of wound healing rates in patients with and without residual osteomyelitis. Forest plot depicting the association of healing in patients with and without residual osteomyelitis following surgical resection. The average OR across studies was calculated as 0.6 (*p* = 0.1, 95% CI 0.3–1.3), indicating that residual osteomyelitis does not have a deleterious effect on wound healing. There was no evidence of heterogeneity, Cochrane *Q* = 1.5 (*p* = 0.7) and *I* ^2^ = 0.0 (95% CI 0.0–70.4).

Four studies^9^, ^12^, ^13^, ^14^ with 97 patients with ROM and 116 patients with no residual bone infection reported healing rates (Figure 5). There was not a significant difference in the proportion of wounds that healed. The fixed effects odds ratio analysis was 0.6 (*p* = 0.1, 95% CI 0.3–1.3). There was no evidence of heterogeneity, Cochrane *Q* = 1.5 (*p* = 0.7) and *I* ^2^ = 0.0 (95% CI 0.0–70.4).

### 3.5 Re‐infection

**FIGURE 6:**
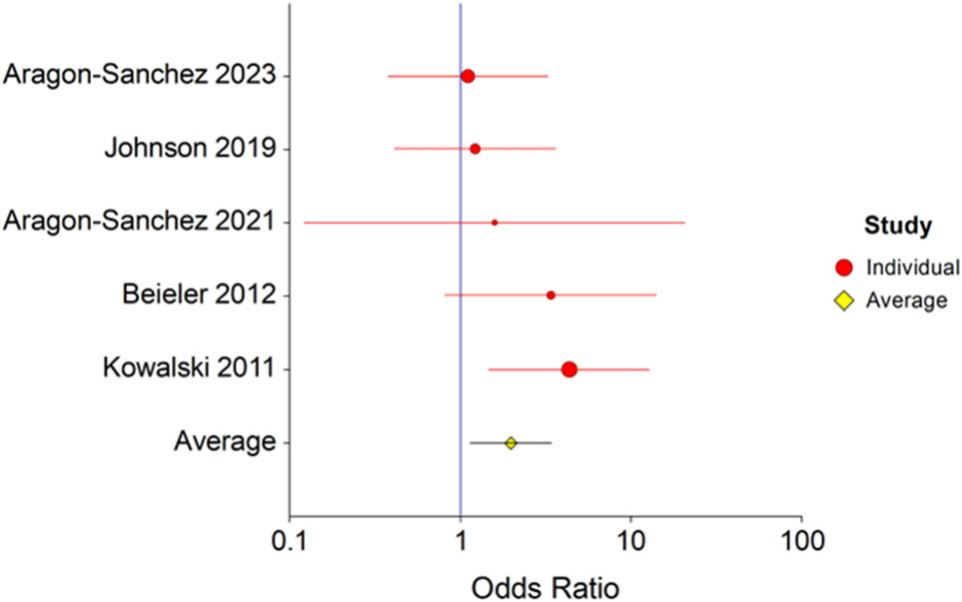
Forest plot of re‐infection in patients with and without residual osteomyelitis. Forest plot depicting re‐infection in patients with residual osteomyelitis following surgical resection. The risk of re‐infection was 2.0 times higher in patients with residual osteomyelitis, (OR = 2.0, *p* = 0.02, 95% CI 1.1–3.4). There was no evidence of heterogeneity, Cochrane *Q* = 4.4 (*p* = 0.35) and *I* ^2^ = 9.11 (95% CI 0.0–81.1).

Five studies^9^, ^13^, ^14^, ^15^, ^16^ with 156 patients with ROM and 183 patients with clean margins reported re‐infection rates (Figure 6). There was a significant difference in re‐infection rates between patients with clean bone margins and ROM. The fixed effects odds ratio analysis was 2.0 (*p* = 0.02, 95% CI 1.1–3.4). There was no evidence of heterogeneity, Cochrane Q = 4.4 (*p* = 0.35) and *I* ^2^ = 9.11 (95% CI 0.0–81.1).

## 4 DISCUSSION

The results of this meta‐analysis demonstrate that patients with ROM have a two‐fold increased risk of re‐infection, and a four‐fold increased risk of amputation compared with patients that had all infected bone surgically resected. In addition, patients with ROM have longer antibiotic treatment. This is the first meta‐analysis to evaluate the results of ROM at the proximal surgical margin in patients with DFO. Conventional wisdom would suggest that ROM would lead to increased risk of re‐infection, lower rates of wound healing, longer duration of antibiotics and more amputations.

Amputation, wound healing, and re‐infection are common outcomes used to define success for the treatment of osteomyelitis.^6^, ^17^ In the studies included in this meta‐analysis, the proportion of wounds that healed in patients with ROM ranged from 55% to 95%. In patients with NROM, the rate of healing ranged from 60% to 100%.^6^ As given in Table 2, only three studies used wound healing as an outcome in evaluating ROM. Most studies that report outcomes in DFO do not report if there is ROM. In these studies, the rate of wound healing is highly variable, reported as high as 86%.^18^, ^19^ Wound healing is a poor surrogate marker for the successful treatment of osteomyelitis because there are many diabetes‐related disease processes and treatments that impact wound healing that are completely unrelated to osteomyelitis.^20^, ^21^ Amputation after the initial treatment for DFO is also used as a measure of success. Patients with ROM were 4.1 times more likely to have an amputation. In the six studies available for analysis, amputation rates for patients with positive bone margins ranged from 16% to 40%. There are many underlying factors that contribute to lower extremity amputation beside osteomyelitis, such as PAD, gangrene, soft tissue infection, chronic kidney disease, socioeconomic status, and access to specialty medical care.^22^, ^23^, ^24^, ^25^ These variables are usually not reported. In DFO studies that were excluded from this meta‐analysis, the incidence of amputation ranged from 36% to 60% (Table 4).^26^, ^27^, ^28^

**TABLE 4:** Summary of outcomes in patients with and without residual osteomyelitis

Variable	Number of studies	Odds ratio	95% CI	*p*‐Value	Cochrane *Q*
Amputation	7	4.3	2.4–7.6	<0.01	1.4
Re‐infection	5	2.0	1.1–3.4	0.02	4.4
Wound healing	4	0.6	0.3–1.3	0.10	1.5

Variable	Number of studies	Mean difference(days)			
Antibiotic duration	4	16.3	11.1–21.1	0.02	4.5

Re‐infection is perhaps the most important outcome to evaluate osteomyelitis treatment. Re‐infection in the five available studies included in the meta‐analysis varied widely and ranged from 14% to 44%. Our results show that re‐infection was two times higher in patients with ROM after bone resection. The importance of evaluating re‐infection is that it can drive re‐hospitalisation which subsequently may lead to more antibiotic administration, and more surgery. In one study, osteomyelitis had a 56% re‐infection rate with nearly all patients requiring re‐admission.^1^

The longer duration of antibiotics may be related to re‐infection or based on the training and belief system of the person prescribing antibiotics. The most common dogma for treating DFO is that it requires 6 weeks of parental antibiotics. However, recent research has compared outcomes based on 3 versus 6 weeks^29^ and 6 versus 12 weeks for DFO.^30^ If the physician making antibiotic decisions embraces the IDSA/IWGDF recommendations,^5^, ^17^ shortening the duration of antibiotic treatment may be due to the treating physician simply follow published recommendations, since there are no clear stopping rules for antibiotics in osteomyelitis.^17^ Aragon‐Sanchez et al.^13^, ^14^ treated patients for the shortest duration whether there was ROM or not.

The strengths of this study include evaluating relevant outcomes important in clinical practice. However, there are some limitations to this meta‐analysis. As in all meta‐analyses, this study was limited by the quality of the original studies. The majority of the studies included were nonrandomized retrospective studies with different inclusion/exclusion criteria. Selection bias may have been introduced through the study design of several studies as patients were only included if they had complete pathology reports or had not deceased within 1 year. Additionally, not all outcomes of interest were available from each publication included in our analysis, which required a pooled analysis from fewer patients thus the full effect may not be fully quantified. Another limitation we identified was that wound healing follow‐up was not clearly defined in most studies. For instance, Aragon‐Sanchez and colleagues reported that the surgical site was assessed two to three times a week until the wound healed.

In addition, there were inconsistent operational definitions for important variables such as how osteomyelitis was initially defined and then how ROM was defined. As given in Table 5, the reference standards varied among studies. Re‐infection was reported in five studies^9^, ^13^, ^14^, ^15^, ^16^ with different definitions between respective studies. Aragon‐Sanchez reported recurrence of infection, which was defined as clinically and/or radiologically diagnosed infection and/or needing antibiotic therapy and/or surgical treatment.^14^ In another study, Aragon‐Sanchez also included wound dehiscence and re‐ulceration as recurrences.^13^ Johnson et al.^9^ and Kowalski et al.^15^ reported re‐infection as relapse which was defined as a confirmed infection of the proximal amputation site via pathological or microbiological examination. Bieler et al.^16^ did not explicitly define infection recurrence. However, all the studies used bone culture and/or histology to define ROM. Some studies have reported poor concordance with bone cultures and histology and poor agreement among pathologists examining the same bone specimens. The outcomes of DFO are difficult to evaluate because there are many other factors in addition to residual bone infection that could have impacted clinical outcomes, such as healing, re‐infection, and amputation. We have not been able to identify a study that identified osteomyelitis as a risk factor for healing, but other co‐morbidities and treatment variables are associated with healing such as PAD, glucose control, and end‐stage renal disease.^31^, ^32^

**TABLE 5:** Summary of the reference standard for studies of residual osteomyelitis.

Reference	Study	No.	OM diagnosis reference standard	Treatment success reference standard
Aragon‐Sanchez et al.^13^	Prospective	93	Clinical signs, probe to bone test, and 2 view radiographs Bone specimen for microbiology and histological analysis	No recurrence within 1 year after the last surgical procedure. (Recurrence defined as clinical signs of infection, requiring antibiotic administration, and/or surgery, and/or re‐admission.) Wound dehiscence and/or re‐ulceration also were considered recurrences
Weng et al.^11^	Retrospective	92	Histopathological report	No same site amputation within 12‐month of index surgery
Aragon‐Sanchez et al.^14^	Retrospective	28	Clinical signs, probe to bone test, and 2 view radiographs Bone specimen for microbiology and histological analysis	No recurrence of infection within 6‐month study period, (recurrence defined as clinically and/or radiologically diagnosed infection and/or needing antibiotic therapy and/or surgical treatment)
Johnson et al.^9^	Retrospective	66	Bone histology report	Complete epithelialization of soft tissue defect and absence of repeat amputation of the index foot
Schmidt et al.^10^	Prospective	72	Clinical symptoms, probe to bone, imaging, laboratory values Bone specimen for microbiology and histological analysis	Not defined
Beieler et al.^16^	Retrospective	50	Histopathologic findings, exposed bone, radiographs or Magnetic Resonance Imaging (MRI)	Not requiring further treatment for DFO
Kowalski et al.^15^	Retrospective	111	Bone culture; bone histology	No infection relapse of proximal amputation site via histopathology or culture
Atway et al.^12^	Retrospective	27	Bone culture, MRI, bone scan, radiography	Amputation site healed without wound dehiscence and full weightbearing

There are several treatments and prevention strategies that impact outcomes that are not addressed in osteomyelitis studies in this meta‐analysis. The first is the type of treatments that are provided after hospital discharge such as off‐loading,^33^ wound debridement and advanced therapies like negative pressure wound therapy or hyperbaric oxygen.^34^ For instance, ulcers treated with total contact casts heal faster with fewer infections compared with other methods to off‐load the foot like shoes or sandals.^35^, ^36^ Multispecialty teams have been shown to improve healing and reduce complications.^37^, ^38^ Second, prevention care after the wound is healed such as bespoke shoes and insoles, regular foot care, and foot‐specific education is never addressed, but has been shown to reduce the incidence of ulcers. For instance, bespoke shoes and insoles have been shown to reduce the incidence of re‐ulceration by half.^39^ Ulceration provides an opening for bacterial infection. It is distinctly uncommon for adults to have a foot infection without a wound. Patients treated without multispecialty care will have fewer wounds that heal and double the incidence of re‐ulceration than patients treated in a more comprehensive group.^37^, ^38^ The cascade of re‐infection, hospitalisation, and amputation would also be significantly higher. Since most patients are not treated in a comprehensive programme, we expect outcomes could be significantly better than reported.

In conclusion, our analysis shows that residual bone infection carries an increased risk of re‐infection and an increased risk of amputation and prolonged antibiotic treatment, but it does not affect wound healing. Future prospective studies should address the limitations of defining residual bone infection and margins with no infection.

## AUTHOR CONTRIBUTIONS

M.C.R. performed data acquisition and initial draft preparation. T.S. and M.A.S. performed data collection. T.L.C. and A.N.T. interpreted and analysed the data. M.J.S. critically reviewed article. L.A.L. formulated study design and wrote the article. All authors discussed the results and contributed to the final article.

## FUNDING INFORMATION

This work was supported by the institutional and departmental funds.

## CONFLICT OF INTEREST STATEMENT

No reported conflicts of interest. All authors have submitted the ICMJE Form for Disclosure of Potential Conflicts of Interest.

## Data Availability

The data that support the findings of this study are available from the corresponding author upon reasonable request.
